# Proto-Oncogene FAM50A Can Regulate the Immune Microenvironment and Development of Hepatocellular Carcinoma In Vitro and In Vivo

**DOI:** 10.3390/ijms24043217

**Published:** 2023-02-06

**Authors:** Xudong Xie, Li Li, Shuai Tao, Mingsheng Chen, Ling Fei, Qunling Yang, Chenlu Huang, Liang Chen

**Affiliations:** 1 Liver Diseases Department, Shanghai Public Health Clinical Center, Fudan University, Shanghai 201508, China; 2 Research Unit, Shanghai Public Health Clinical Center, Fudan University, Shanghai 201508, China; 3 Research Center for Biomaterials, Shanghai Public Health Clinical Center, Fudan University, Shanghai 201508, China

**Keywords:** hepatocellular carcinoma, FAM50A, prognostic value, immune cell infiltration, stemness degree, epithelial–mesenchymal transition, cell cycle, apoptosis, malignancy, xenotransplanted tumor

## Abstract

Hepatocellular carcinoma (HCC) is a vital global health problem. The characteristics are high morbidity, high mortality, difficulty in early diagnosis and insensitivity to chemotherapy. The main therapeutic schemes for treating HCC mainly include Tyrosine kinase inhibitors represented by sorafenib and lenvatinib. In recent years, immunotherapy for HCC has also achieved certain results. However, a great number of patients failed to benefit from systemic therapies. FAM50A belongs to the FAM50 family and can be used as a DNA-binding protein or transcription factor. It may take part in the splicing of RNA precursors. In studies of cancer, FAM50A has been demonstrated to participate in the progression of myeloid breast cancer and chronic lymphocytic leukemia. However, the effect of FAM50A on HCC is still unknown. In this study, we have demonstrated the cancer-promoting effects and diagnostic value of FAM50A in HCC using multiple databases and surgical samples. We identified the role of FAM50A in the tumor immune microenvironment (TIME) and immunotherapy efficacy in HCC. We also proved the effects of FAM50A on the malignancy of HCC in vitro and in vivo. In conclusion, we confirmed that FAM50A is an important proto-oncogene in HCC. FAM50A acts as a diagnostic marker, immunomodulator and therapeutic target for HCC.

## 1. Introduction

HCC is the sixth most common cause of cancer (4.7%) and the third leading cause of cancer death (8.3%) worldwide [1]. The main risk factors of HCC principally comprise hepatitis virus, non-viral hepatitis, aflatoxin and alcoholism [2]. Methods for diagnosing and treating HCC have improved in recent years, including imageological examination of the liver and serum alpha-fetoprotein (AFP) [3]. A detection kit based on seven microRNAs [4] and the GALAD diagnostic model [5] has also been used. However, due to the insidious onset of HCC, no more than 30% of patients can be diagnosed at an early stage and receive radical treatment. For those who get advanced HCC, systematic antitumor therapies can only control the progression and prolong the survival time. According to statistics, in the United States [6] and Asia [7], the five-year survival rate of HCC patients is only 15–38%. The prognosis of HCC still faces enormous challenges.

The most useful drugs for HCC before were multi-target receptor kinase inhibitors, represented by sorafenib [8,9], lenvatinib [10] and donafenib [11], which are used in the systemic treatment of HCC. In recent years, immunotherapy has shown tremendous progress [12,13,14]. Immunotherapy combined with targeted therapy for HCC has also shown outstanding results in clinical studies [15,16,17]. However, only a few patients with HCC can benefit from systematic treatment. This may be related to the adverse drug reactions [18,19], drug resistance [20] and tumor microenvironment (TME) [21] of patients. Therefore, it is necessary to find new biomarkers and therapeutic targets for HCC.

The Family with Sequence Similarity (FAM) contains a collection of multiple genes with similar sequences. These genes play important roles in a variety of diseases. For example, FAM20C is widely expressed in a variety of cancer tissues, and it has shown to be tumorigenic in humans. FAM20C expression was positively correlated with immune infiltration and multiple immune cells [22]. The FAM50 gene family consists of two members, namely FAM50A and FAM50B. FAM50A is a basic protein that contains a nuclear localization sequence and can act as a DNA binding protein or transcription factor. The cytogenetic location of FAM50A is human chromosome Xq28. FAM50A may act as a kind of splicing factor in the splicing of RNA precursors. Studies have shown that FAM50A is associated with the development of X-chromosome-related intellectual disability [21]. FAM50A was found to be involved in the regulation of lncRNA CD36-005 on the endometrial stromal cells of rats with polycystic ovary syndrome [23]. During the study of tumors, the researchers found that, in medullary breast cancer tissue samples with abundant lymphocytic infiltration, the expression of FAM50A in immune cells was significantly higher than that in both cancer and non-cancer cells [24]. In the study of peripheral blood mononuclear cells (PBMC) from patients with chronic lymphoblastic leukemia (CLL), FAM50A was considered to be a candidate driver gene for the recurrence of CLL. This is related to immunotherapy [25]. These studies suggest that FAM50A plays an important role in tumorigenesis and development. It may participate in tumor immune response. FAM50A has the potential to be a target for immunotherapy. Wang et al. first identified the splicing factor FAM50A as a risk factor for HCC in their study [26]. However, there are few studies on FAM50A. The effects of FAM50A on the pathological and immune mechanisms of HCC are still unclear.

We collected data from The Cancer Genome Atlas (TCGA) database, the Gene Expression Overview (GEO) database, the University of Alabama at Birmingham Cancer data analysis Portal (UALCAN) database and surgical samples from 180 HCC patients and 33 patients with benign liver diseases treated in the Affiliated Cancer Hospital of Nantong University from 2010 to 2017. We verified the expression of FAM50A in liver tissues of HCC patients and analyzed the correlation with prognosis. Secondly, the effects of FAM50A on TIME and immunotherapy response were analyzed in combination with the Tumor Immune Estimation Resource (TIMER) database. We knocked down and overexpressed FAM50A in HCC cell lines. Combined with the subcutaneous xenotransplantation of HCC models in nude mice, we identified the role of FAM50A in the occurrence and development of HCC. In conclusion, this research confirmed the important role of FAM50A in the development, TIME and prognosis of HCC.

## 2. Results

### 2.1. Expression of FAM50A in HCC

**Figure 1:**
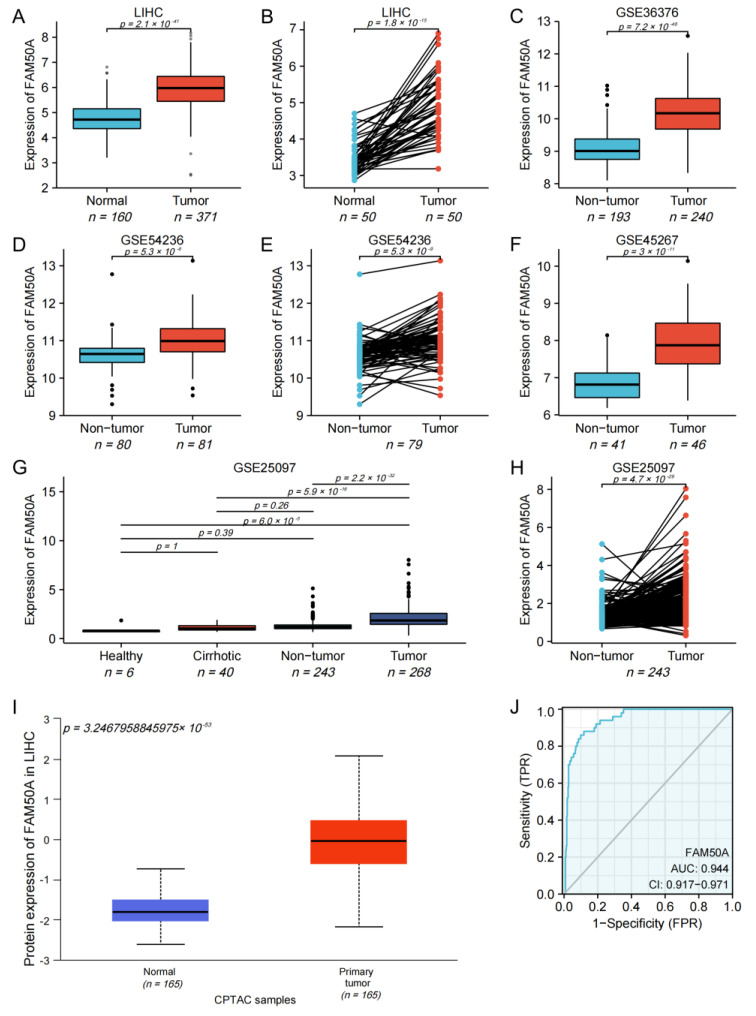
Expression and diagnostic efficacy of FAM50A in HCC. R software was used to perform Student’s *t* tests on data from multiple databases. The mRNA and protein levels of FAM50A in HCC tissues were higher than those in benign liver tissues. (**A**) Grouping comparison of RNA data from TCGA. (**B**) Paired comparison of RNA data from TCGA. (**C**) Grouping comparison of RNA data from GSE36376. (**D**) Grouping comparison of RNA data from GSE54236. (**E**) Paired comparison of RNA data from GSE54236. (**F**) Grouping comparison of RNA data from GSE45267. (**G**) Grouping comparison of RNA data from GSE25097. (**H**) Paired comparison of RNA data from GSE25097. (**I**) Grouping comparison of protein expression by UALCAN. (**J**) ROC analysis showed that the AUROC of FAM50A for HCC patients in the TCGA database was 0.944 and the CI was 0.917–0.971.

We found that the mRNA expression of FAM50A in 371 HCC tissues was significantly higher than that in 160 normal tissues (Figure 1A). The mRNA expression of FAM50A in cancerous tissues of 50 HCC patients was significantly higher than that in paracancerous tissues (Figure 1B). For HCC patients with different pathological stages, T stages and vascular invasion, the mRNA expression of FAM50A was also significantly different (Supplementary Figure S1A–C). The mRNA expression of FAM50A was also verified by different GEO datasets (GSE36376, GSE54236, GSE45267 and GSE25097). We found that the expression of FAM50A in HCC tissues was significantly higher than that in normal tissues and cirrhotic tissues. The datasets GSE54236 and GSE25097 showed that the expression of FAM50A in HCC tissues was significantly higher than that in paracancerous tissues (Figure 1C–H). We used UALCAN to determine that the protein level of FAM50A was significantly higher in HCC tissues than in normal tissues (Figure 1I). The receiver operating characteristic (ROC) analysis showed that the area under ROC (AUROC) of FAM50A in HCC patients was 0.944, and the 95% confidence interval (CI) was 0.917–0.971. In conclusion, the expression of FAM50A is higher in HCC tissues than in normal tissues, and it has the potential to act as a diagnostic marker for HCC.

### 2.2. Prognostic Value of FAM50A in HCC

We assessed whether FAM50A and other clinical parameters were independent risk factors for HCC. Based on univariate COX regression analysis, we found that the pathological stage, T stage, M stage, tumor status and FAM50A expression were significantly correlated with the overall survival (OS) of patients with HCC (Table 1). Disease-specific survival (DSS) was significantly correlated with Child–Pugh grade, pathological stage, T stage, M stage, prothrombin time and the expression of FAM50A (Supplementary Table S1). The results of multivariate COX regression analysis indicate that FAM50A expression and tumor status were independent predictors of OS for HCC patients (Table 1). The independent predictors of DSS for HCC patients were FAM50A expression and Child–Pugh grade (Supplementary Table S1).

### 2.3. Effect of FAM50A on Survival of HCC Patients

**Figure 2:**
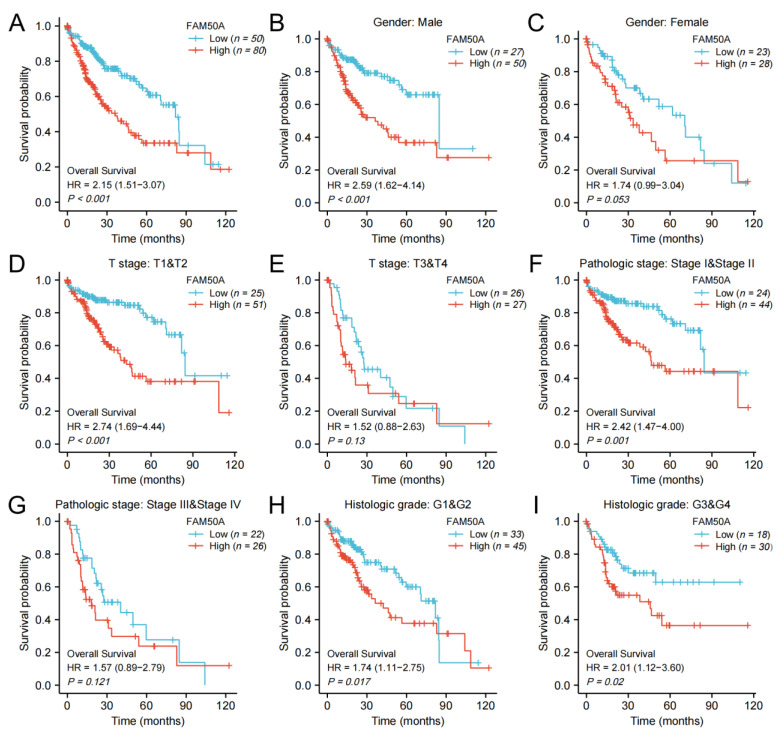
Kaplan–Meier survival analysis. We analyzed the effects of FAM50A on OS in HCC patients with the help of information from TCGA. (**A**) All HCC patients. (**B**) Male patients. (**C**) Female patients. (**D**) T stage (T1 and T2). (**E**) T stage (T3 and T4). (**F**) Pathologic stage I and II. (**G**) Pathologic stage III and IV. (**H**) Histologic grade 1 and 2. (**I**) Histologic grade 3 and 4.

We calculated the effect of FAM50A on the survival of HCC. In general, Kaplan–Meier survival analysis showed that patients with low expressions of FAM50A have better outcomes in terms of OS and DSS (Figure 2A, Supplementary Figure S2A). For male patients, patients with T stage T1 and T2, and patients with pathologic stage I and II, low expression of FAM50A predicted better OS and DSS, respectively, than the higher-expression group (Figure 2B,D,F, Supplementary Figure S1B,D,F). In female patients, patients with T stage T3 and T4, and patients with pathological stage III and IV, there was no significant correlation of FAM50A with OS and DSS (Figure 2C,E,G, Supplementary Figure S2C,E,G). For patients with different histologic grades, lower expression of FAM50A predicted better OS, while FAM50A did not affect DSS (Figure 2H,I, Supplementary Figure S2H,I). Overall, the expression of FAM50A has an important prognostic value for HCC patients.

### 2.4. Relationship of FAM50A and Immune Cell Infiltration in HCC

**Figure 3:**
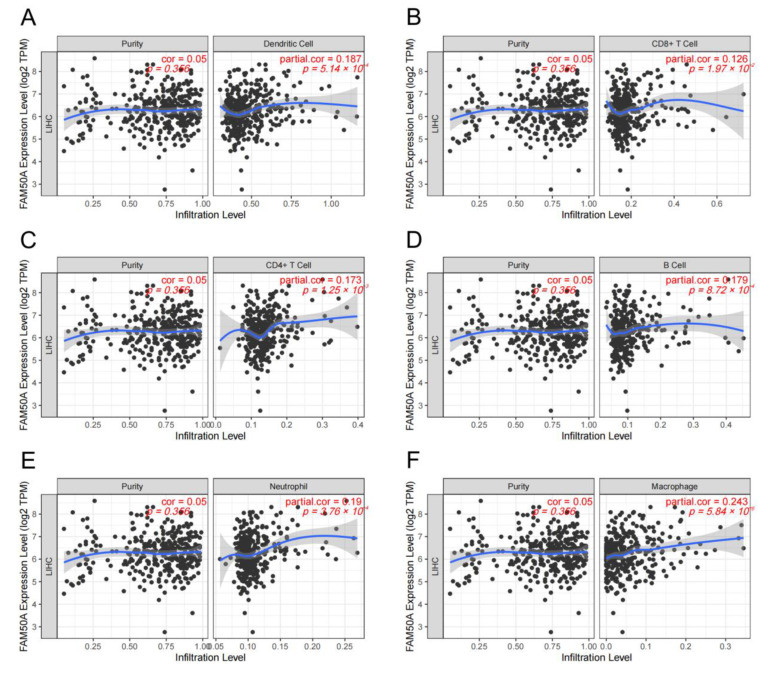
Correlation between FAM50A and immune cell infiltration levels in HCC. We analyzed the correlation between the expression of FAM50A and the infiltration of multiple immune cells in the TIMER database. (**A**) Dendritic cells. (**B**) CD8+ T cells. (**C**) CD4+ T cells. (**D**) B cells. (**E**) Neutrophils. (**F**) Macrophages.

A great number of immune cells exist both inside and outside the tumor, called TIME. These immune cells undergo complex interactions with the tumor cells. The adequate analysis of the particular TIME is important to predict and guide immunotherapy. We calculated the relationship between FAM50A and immune cell infiltration in HCC using the TIMER platform. It showed that there was a significant positive correlation between FAM50A and immune cells (dendritic cells, CD8+ T cells, CD4+ T cells, B cells, neutrophils and macrophages) (Figure 3A–F). The expression of FAM50A was positively correlated with that of immune cell markers (Supplementary Table S2). These results suggest that FAM50A occupies an important position in the immune infiltration of HCC.

### 2.5. Corrections between FAM50A and Stemness of HCC Cells

**Figure 4:**
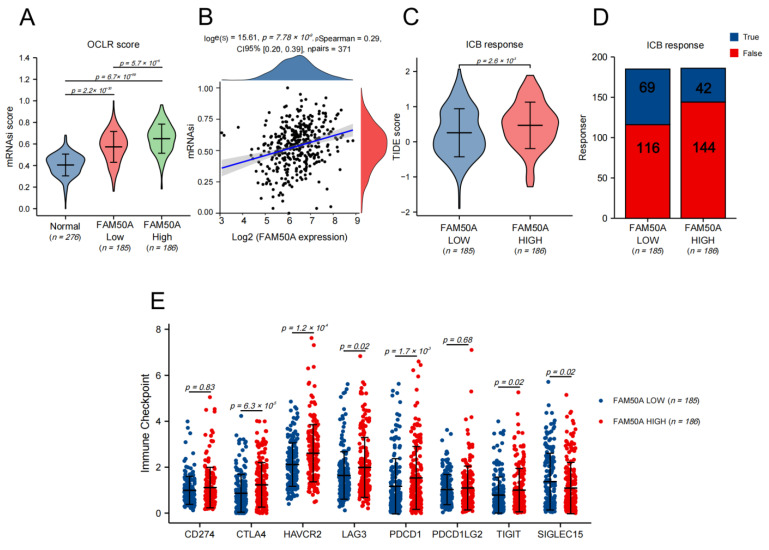
The effects of FAM50A on stemness degree, immunotherapy response and the expression of the immune checkpoint in HCC. We used the OCLR algorithm to calculate the stemness degree and the tumor immune dysfunction and exclusion (TIDE) algorithm to evaluate the response to immune checkpoint blockade (ICB). (**A**) The stemness degrees of 276 normal liver tissues, 185 FAM50A low-expression tissues and 186 FAM50A high-expression tissues. (**B**) Correlation between FAM50A and stemness degree of 371 HCC patients. (**C**) The TIDE scores of 185 FAM50A low-expression tissues and 186 FAM50A high-expression tissues. (**D**) Number of patients with low expressions of FAM50A and patients with high expressions of FAM50A who responded to immunotherapy. (**E**) Correlation between FAM50A and immune checkpoint expression in HCC.

The characteristics of cancer include the progressive loss of the differentiated phenotype and the acquisition of stem cell-like features, which include the self-renewal and unlimited proliferation abilities for maintaining tumor growth, the movement and migration abilities to promote tumor metastasis, and an insensitivity to anti-tumor therapy. We used the TCGA database to calculate the stemness index using the one-class logistic regression (OCLR) algorithm so as to assess the stemness degree of the HCC samples. We found that the stemness degree of HCC tissues was significantly higher than that of normal liver tissue. The stemness degree of the FAM50A-high group was significantly higher than the FAM50A-low group (Figure 4A). The stemness degree of HCC was positively correlated with the expression of FAM50A (Figure 4B). These results indicate that patients with high FAM50A expression are more likely to develop adverse consequences, such as rapid tumor progression, tumor metastasis and tumor recurrence.

### 2.6. Correlation between FAM50A and Immunotherapy

Tumor immunotherapy is a kind of therapeutic method that aims to cure tumors by restarting the tumor immune cycle and restoring the anti-tumor immune response. For HCC, the current first-line treatment regimen is a combination of atezolizumab and bevacizumab [15]. We assessed the response to ICB therapy in HCC using the TIDE algorithm. The results show that the FAM50A low-expression group had a better ICB response than the high-expression group (Figure 4C). Patients with low FAM50A expression are more likely to benefit from immunotherapy (Figure 4D). We went on to analyze the expressions of immune checkpoint markers, finding that CTLA4, HAVCR2, LAG3, PDCD1, and TIGIT were elevated in the FAM50A high-expression group compared to the FAM50A low-expression group. The expression trend of SIGLEC15 is opposite to that stated above. Theoretically, we can use the corresponding immunotherapy regimen according to FAM50A expression.

### 2.7. Effects of FAM50A on HCC In Vitro

**Figure 5:**
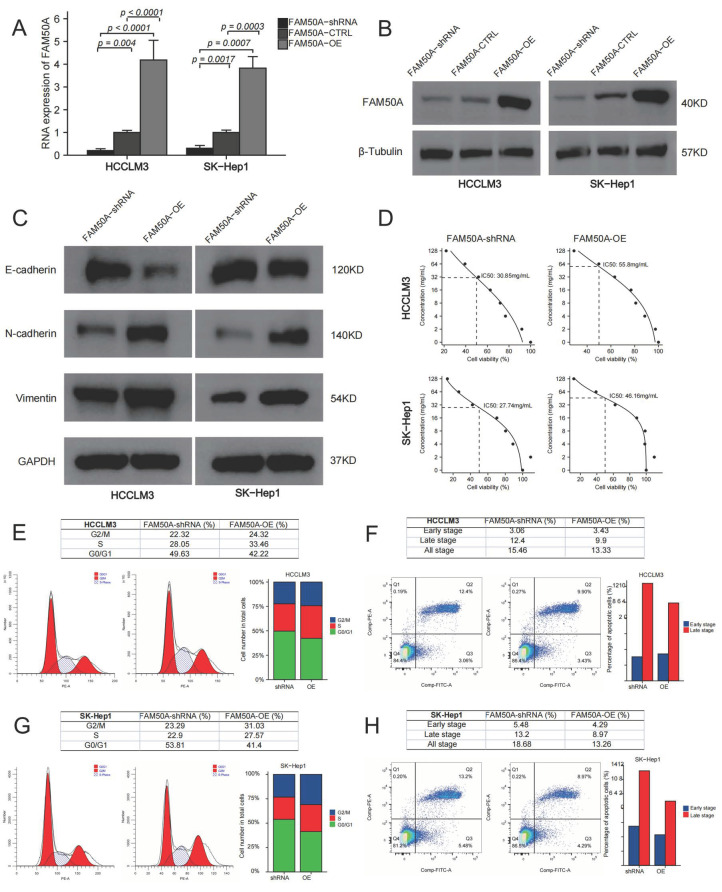
Effects of FAM50A on the function of HCC cells. We knocked down and overexpressed FAM50A expression in HCCLM3 and SK-Hep1 cells to explore the efficacy of FAM50A on EMT, drug susceptibility, cell cycle and apoptosis of HCC cells. (**A**) The mRNA expression of FAM50A detected by qRT-PCR. (**B**) The protein expression of FAM50A detected by Western blot and β-tubulin was used as the internal reference. (**C**) The protein expression of E-cadherin, N-cadherin and vimentin detected by Western blot and GAPDH was used as the internal reference. (**D**) IC_50_ of different cells: FAM50A-shRNA-treated HCCLM3 cells (30.85 mg/mL), FAM50A-OE HCCLM3 cells (55.8 mg/mL). FAM50A-shRNA-treated SK-Hep1 cells (27.74 mg/mL), FAM50A-OE SK-Hep1 cells (46.16 mg/mL). (**E**,**F**) The cell cycle of FAM50A-shRNA-treated cells and FAM50A-OE cells was determined by flow cytometry, and we got the proportion of cells at different periods. (**G**,**H**) Apoptosis of FAM50A-shRNA-treated cells and FAM50A-OE cells was measured by flow cytometry, and we got the proportions of early- and late-stage apoptosis.

We overexpressed and downregulated the expressions of FAM50A in two HCC cell lines (HCCLM3 and SK-Hep1). Quantitative real-time polymerase chain reaction (qRT-PCR) and Western blot were applied to test the expression of FAM50A. The mRNA and protein expressions of the control were significantly lower than those of FAM50A-OE-treated cells, while they were higher than those of FAM50A-short hairpin RNA (shRNA)-treated cells (Figure 5A,B).

#### 2.7.1. Effects of FAM50A on Epithelial–Mesenchymal Transition (EMT)

EMT refers to the biological phenomenon whereby epithelial cells transform into cells with a stromal phenotype. EMT is closely related to tumor infiltration and metastasis. We found that FAM50A-shRNA-treated cells had increased expression of E-cadherin, and decreased expressions of N-cadherin and vimentin, compared to FAM50A-overexpressing (FAM50A-OE) cells. This suggests that the downregulation of FAM50A suppressed the development of EMT, which may inhibit tumor infiltration or metastasis (Figure 5C).

#### 2.7.2. FAM50A Regulates the Sensitivity of HCC Cells to Lenvatinib

Lenvatinib is a kind of multi-target receptor tyrosine kinase that can suppress multiple targets, such as the vascular endothelial growth factor receptor (VEGFR). We cultured FAM50A-shRNA-treated cells and FAM50A-OE cells with different concentrations of lenvatinib. The half-maximal inhibitory concentration (IC_50_) was calculated by detecting the cell activity. The IC_50_ was 30.85 mg/mL in FAM50A-shRNA-treated HCCLM3 cells, and 55.8 mg/mL in FAM50A-OE HCCLM3 cells. The IC_50_ was 27.74 mg/mL in FAM50A-shRNA-treated SK-Hep1 cells and 46.16 mg/mL in FAM50A-OE SK-Hep1 cells (Figure 5D). This indicates that decreased FAM50A expression makes HCC cells more sensitive to lenvatinib. This suggested that FAM50A had the potential to regulate the resistance of HCC to lenvatinib in vivo.

#### 2.7.3. Effect of FAM50A on Cell Cycle and Apoptosis of HCC

We used flow cytometry (FCM) to analyze the cell cycle and apoptosis of FAM50A-shRNA-treated and FAM50A-OE HCC cells. FAM50A-shRNA-treated cells had a lower proportion of S phase cells and a higher proportion of G0/G1 cells than did FAM50A-OE cells (Figure 5E,F). The proportion of apoptotic cells increased in FAM50A-shRNA-treated cells; among the main elevated cells were late apoptotic cells (Figure 5G,H). This means the downregulation of FAM50A not only inhibited cell proliferation and cell viability, but also promoted apoptosis.

#### 2.7.4. Effects of FAM50A on the Malignancy of HCC Cells

**Figure 6:**
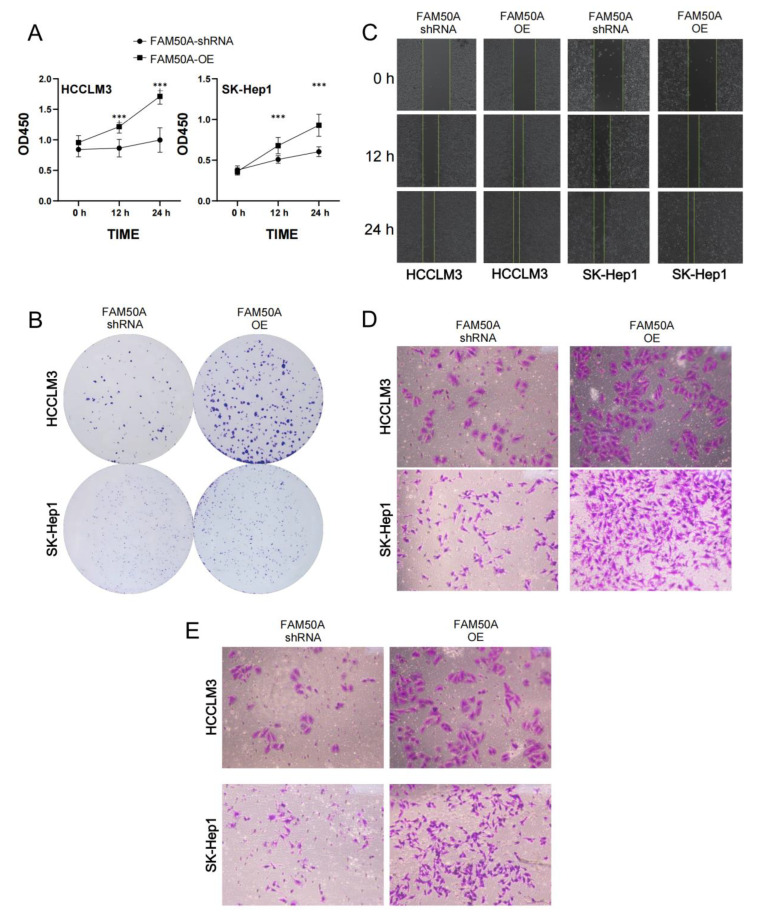
Effects of FAM50A on the proliferation, migration and invasion of HCC cells. (**A**) The proliferation capacity of FAM50A-shRNA-treated cells and FAM50A-OE cells, determined by Cell Counting Kit-8 (CCK-8) assay. The broken line graph shows the cell growth. (**B**) The proliferation ability of FAM50A-shRNA-treated cells and FAM50A-OE cells was determined by colony formation assay. Cell colonies were stained with crystal violet. (**C**) The cell migration ability of FAM50A-shRNA-treated cells and FAM50A-OE cells was determined by wound healing assay. The blank space between the two lines reflects the extent of cell migration. (**D**) The cell migration ability of FAM50A-shRNA-treated cells and FAM50A-OE cells was determined by the transwell assay. The cells were stained with crystal violet. (**E**) The invasion ability of FAM50A-shRNA-treated cells and FAM50A-OE cells was determined by transwell assay with the addition of matrix gel. The cells were stained with crystal violet. *** *p <* 0.001.

We investigated the proliferation of FAM50A-shRNA-treated and FAM50A-OE HCC cells using the CCK8 assay and clone formation assay. We found that the viability of FAM50A-shRNA-treated cells was lower than that of FAM50A-OE cells after being cultured for 12 h and 24 h. (Figure 6A). The cell colonies formed after 14 days of culture. The clone number of FAM50A-shRNA-treated cells was significantly less than that of FAM50A-OE cells (Figure 6B). It appears that the high expression of FAM50A can promote tumor proliferation.

The wound healing assay and transwell migration tests can reflect the migration ability of HCC. The wound was marked when the cell density reached 100%. FAM50A-shRNA-treated cells showed a significantly lower healing rate than FAM50A-OE cells (Figure 6C). After 30 h of cell culturing in the chamber, we found that the number of FAM50A-shRNA-treated cells passing through the chamber was significantly lower than that of the FAM50A-OE cells (Figure 6D). These results demonstrate that the downregulation of FAM50A inhibited the migration of HCC cells.

Cell invasion requires cells to cross the extracellular matrix (ECM) or the basement membrane matrix (BMM), in a process whereby cells need to first hydrolyze ECM or BME with enzymes. We performed the transwell invasion assay with the combination of a transwell chamber and matrix gel to test the invasion ability of cells. The process of culturing cells was performed on the transwell migration assay. We found that FAM50A-shRNA-treated cells were significantly weaker than FAM50A-OE cells in their ability to penetrate the chamber through the hydrolyzed matrix gel (Figure 6E). This reflects that the high expression of FAM50A promotes the invasive capacity of HCC cells.

In conclusion, we found that highly expressed FAM50A can promote the transformation of HCC cells from epithelial cells to mesenchymal cells in vitro. FAM50A promoted tumor infiltration or metastasis, and it reduced the sensitivity to lenvatinib. It also inhibited cell apoptosis, and promoted cell division, proliferation and migration and the invasion of HCC cells. All these suggest that FAM50A acts as a cancer-inducer in HCC.

### 2.8. Effect of FAM50A on HCC In Vivo

**Figure 7:**
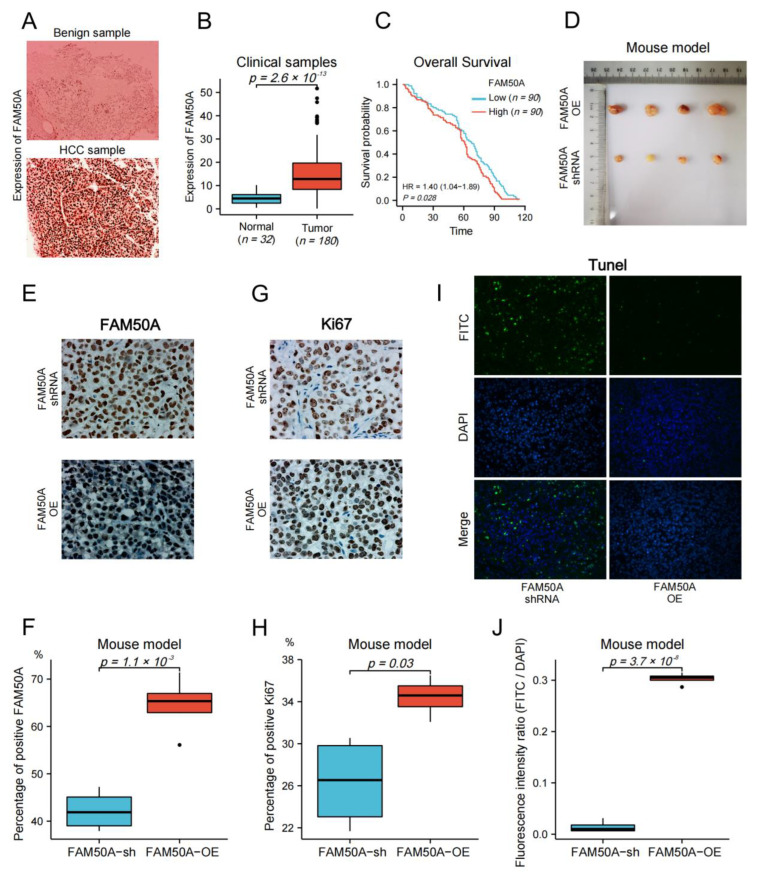
Effect of FAM50A on HCC in vivo. (**A**) The protein expression of FAM50A in benign liver tissues and HCC tissues was detected by IHC. (**B**) The software Image J was used to quantitatively analyze the expression of FAM50A in tissues. The expression of FAM50A in 180 HCC tissues was significantly higher than that in 32 benign liver tissues. (**C**) K-M analysis of 180 HCC patients showed that half of the patients with low FAM50A expression had better OS than the other half of the patients with low FAM50A expression. (**D**) Subcutaneous xenograft tumors formed in nude mice by FAM50A-shRNA-treated cells and FAM50A-OE cells. (**E**,**F**) The protein expression of FAM50A in subcutaneous xenograft tumors of nude mice was detected by IHC and analyzed by Image J. (**G**,**H**) The expression of Ki67 in subcutaneous xenograft tumors of nude mice was detected by IHC and analyzed by Image J. (**I**,**J**) TUNEL intensity in subcutaneous xenograft tumors of nude mice was measured by IF and analyzed by Image J.

We collected surgical samples from 32 patients with benign liver disease, and 180 HCC patients that received surgical treatment at the Affiliated Cancer Hospital of Nantong University from 2010 to 2017. We followed up on the survival time of HCC patients until April 2022. Immunohistochemistry (IHC) was applied to detect the protein expression in tissues. The expression of FAM50A in benign tissues was significantly lower than that in malignant tissues (Figure 7A,B). We evenly divided the HCC patients into high- and low-expression groups according to the expression of FAM50A. Then, we carried out the survival analysis according to the prognostic information of patients. The Kaplan–Meier survival curves show that the OS of the FAM50A low-expression group was significantly better than that of the high-expression group (Figure 7C).

We subsequently subcutaneously injected FAM50A-shRNA-treated and FAM50A-OE HCCLM3 cells into BALB/c nude mice, and then xenotransplanted tumors were later grown subcutaneously. The sizes of tumors from FAM50A-shRNA-treated cells were significantly smaller than tumors from FAM50A-OE cells (Figure 7D). Ki67 is a nuclear antigen that is associated with cell proliferation and is positively correlated with the malignancy of cancer. IHC showed that the expression of FAM50A and Ki67 in tumors from FAM50A-shRNA-treated cells was significantly lower than that in tumors from FAM50A-OE cells (Figure 7E–H). This means the elevated expression of FAM50A promotes tumor growth in vivo. The apoptosis rate was detected by TUNEL. Apoptotic cells were labeled with fluorescein isothiocyanate (FITC) by immunofluorescence (IF) staining, and all nuclei were labeled with DAPI. The extent of apoptosis was evaluated by the fluorescence intensity ratio of FITC to DAPI. Apoptosis was more frequent in tumors from FAM50A-shRNA-treated cells in tumors from FAM50A-OE cells (Figure 7I,J). This result shows that the high expression of FAM50A inhibited tumor cell apoptosis in vivo.

## 3. Discussion

HCC is one of the most important global health problems. It has high incidence and mortality rates. Given that the mortality rate is higher than the incidence rate, it is apparent that the prognosis of HCC is poor, which results in a severe burden on human health. In terms of pathogenesis, the relative peak population of diagnosing HCC is men aged 60 to 70 years [27]. As age increases, the incidence of patients improves accordingly. HCC is a kind of malignancy related to multiple risk factors. Hepatitis viruses are important risk factors for chronic liver disease and cirrhosis, which can cause HCC [28]. The hepatitis B virus vaccine [29] and antiviral drugs [30] have been widely used. In particular, drugs against the hepatitis C virus can almost achieve a healing effect [31]. These measures partly control HCC induced by the hepatitis viruses.

However, the incidence of metabolic diseases, including obesity and type II diabetes, has risen dramatically. The main manifestation of metabolic diseases in the liver is nonalcoholic fatty liver disease (NAFLD) [32]. The sustained progression of NAFLD may cause fibrosis, cirrhosis and even HCC [33,34]. As the global incidence is 25% [35], NAFLD has become one of the most common chronic liver diseases. Thus, although the prevalence of virus-caused HCC has been slightly relieved, the incidence rate of NAFLD-related HCC is still increasing. Some other causes of HCC include alcohol consumption, aflatoxin, parasites, etc. The morbidity and mortality of HCC continue to rise. Studies have shown that in some countries, such as the United States, the incidence of HCC will continue to rise until 2030 [36].

Another important reason for the poor prognosis of HCC is that most patients have reached the middle or advanced stage at the time of diagnosis, which prevents them from undergoing curative surgical treatment. Most patients can only receive comprehensive therapy, such as chemotherapy, targeted therapy and immunotherapy, to prolong their life [37]. While the combination of targeted therapy and immunotherapy has achieved some efficacy [15], due to the differences between patients in terms of TME, drug sensitivity and tolerance to adverse drug reactions, not all patients can benefit from comprehensive treatment. Therefore, we need to comprehensively evaluate the TME and sensitivity to drugs of each patient in the process of developing a treatment plan.

In this study, we investigated the role of FAM50A in HCC. FAM50A is a member of the FAM50 family and is mainly present in the nucleus. Researchers initially found that it was associated with diseases such as Armfield syndrome and intellectual developmental disorder. In the study of tumors, researchers found the pro-cancer role of FAM50A in medullary breast carcinoma [24], chronic lymphoblastic leukemia and other tumors [25].

We confirmed that the expression of FAM50A in HCC tissues was significantly higher than that in normal tissues through the TCGA database, multiple GEO datasets and surgical samples. The prognosis of HCC patients with a low expression of FAM50A was significantly longer than that of patients with a high expression of FAM50A. FAM50A had both diagnostic value and independent prognostic value in HCC. Chen’s group demonstrated that the expression of PDZD11 increased in tumor tissues and was associated with poor prognosis. The expression of PDZD11 was positively correlated with the infiltration of six immune cells (B cell, CD4+ T cell, CD8+ T cell, macrophage, neutrophil, and dendritic cell) in HCC. In addition, they confirmed that PDZD11 expression was significantly positively correlated with FAM50A expression [38]. It can be inferred that the expression of FAM50A may also regulate the immune status of HCC patients. In our study, we demonstrated that FAM50A was significantly associated with immune cell infiltration, response to immunotherapy and immune checkpoint expression. These findings indicate the regulatory effect of FAM50A on the TIME of HCC. We also demonstrated the effects of FAM50A on proliferation, migration, invasion, EMT, cell cycle, apoptosis and sensitivity to lenvatinib in vitro. Then, we identified the role of FAM50A in vivo via subcutaneous xenograft tumors in nude mice. In conclusion, we clarified the pro-cancer role of FAM50A in HCC from multiple perspectives. It is suggested that FAM50A can be used as a diagnostic marker and therapeutic target for HCC.

However, there are some limitations in this study. While we have clarified the role of FAM50A in HCC, we did not extensively describe the mechanism of FAM50A in HCC, which is a work in progress. In addition, we are also investigating the clinical application of FAM50A as a therapeutic target for treating HCC.

## 4. Materials and Methods

### 4.1. Data and Tissue Samples Collection

RNA seq data of HCC patients were downloaded from the TCGA database (https://portal.gdc.cancer.gov/) (Accessed on 16 February 2022). The prognostic information was downloaded from existing studies [39]. The GSE36376, GSE54236, GSE45267, and GSE25097 datasets were downloaded from the GEO database.

Surgical samples were taken from 180 HCC patients and 32 patients with benign liver diseases who were treated at the Affiliated Cancer Hospital of Nantong University from 2012 to 2017. None of the patients received radiotherapy or chemotherapy before surgery. All tissues were pathologically examined to determine their properties, and then stored in wax blocks.

The UALCAN website was used to analyze the protein expression of FAM50A in normal liver tissues and HCC tissues. The relevant data came from the Clinical Proteomic Tumor Analysis Consortium (CPTAC). *p* < 0.05 indicates statistical significance.

### 4.2. Cell Culture

The HCC cell lines HCCLM3 and SK-HEP1 were purchased from the BeNa Culture Collection (Beijing, China). HCCLM3 cells were cultured in Dulbecco’s modified Eagle’s medium (DMEM) (Corning, NY, USA) and SK-Hep1 cells were cultured in RPMI-1640 medium (Corning, NY, USA). The media were supplemented with 10% fetal bovine serum (FBS) (Lonsera, Shanghai, China). The cells were cultured in an incubator at 37 ℃ and 5% CO_2_.

### 4.3. Bioinformatics Analysis by R Software

The RNA sequencing data of HCC obtained from TCGA were converted from the fragments per kilobase million (FPKM) format to the transcripts per million (TPM) format and converted by log2. The differential expression of genes between different groups was analyzed by the Wilcoxon rank sum test and the R package ggplot2 was applied to visualize the results. Pearson analysis was employed to examine the association of FAM50A with immune cell markers in HCC. The ROC analysis was conducted by the R package pROC and the results were visualized by the R package ggplot2. The prognostic information of patients with HCC was analyzed by COX regression with the survival package. The Kaplan–Meier survival estimate between the low- and high-expression groups of FAM50A in HCC was also performed by the survival package. The survminer package was applied to visualize the K-M curves.

The stemness degree was determined according to the OCLR algorithm constructed by Malta et al. [40], and the mRNAsi was calculated from the RNA sequencing data of HCC from TCGA and the corresponding clinical information.

The ICB response was calculated according to the TIDE algorithm using the RNAseq data and the corresponding clinical information of HCC from TCGA. The ggpubr package was used to predict the potential immunotherapy response and the results were visualized by the R software package ggplot2 [41].

### 4.4. Analysis of Immune Infiltration by TIMER

The TIMER database can be applied to systematically analyze immune cell infiltration in different kinds of cancers [42]. Through the TIMER algorithm, we analyzed the correlation between FAM50A and six immune-infiltrating cells (B cells, CD4+ T cells, CD8+ T cells, neutrophils, macrophages and dendritic cells).

### 4.5. Plasmids, Lentiviruses, and Regulation of Gene Expression

We synthesized FAM50A-specific shRNA plasmid (5′-AAGGGAAGAGATCACCACGAA-3′) and overexpression plasmid from Genewiz (Suzhou, China). A plasmid that contains nonsense sequences was used as a control. With the help of the EZ Trans cell transfection agent (Life-iLab Shanghai, China), the above plasmids were co-transfected into 293T cells with packaging plasmid psPAX2 and envelop plasmid pMD2.G. The supernatant of the cell culture medium was replaced at 12 h after transfection. After 72 h, the supernatant was collected and concentrated to obtain lentiviruses. Lentiviruses and transfection reagents were added to HCC cells in culture. The cell supernatant was replaced 12 h after infection and the cells were treated with puromycin (Beyotime Biotechnology Beijing, China) 48 h later to obtain transfected cell lines.

### 4.6. RNA Extraction and qRT-PCR

Total RNA was extracted from cells according to the instructions of the RNA Isolation Kit (Tiangen, Beijing, China). Then, 2 μg of total RNA was reverse transcribed into cDNA by the PrimeScript RT reagent kit for qRT-PCR (TaKaRa, Shiga, Japan). The reaction system of qRT-PCR was performed according to the instructions of the TB Green Premix Ex Taq™ II kit (TaKaRa, Shiga, Japan). GAPDH acted as the internal reference. The primers used were as follows: FAM50A forward 5′-TTCCGGGAGCTGGGAGATAA-3′, reverse 5′-CTCACCACCCACTCCAAGTC-3′, GAPDH forward 5′-CGGAGTCAACGGATTTGGTCGT-3′, reverse 5′-TCTCAGCCTTGACGGTGCCA-3′. The relative expression of genes was calculated by the 2^−∆∆CT^ method [43].

### 4.7. Western Blot Analysis

Total cellular proteins were extracted by RIPA lysis buffer (NCM Biotech, Suzhou, China) combined with protease inhibitor and phosphatase inhibitor (NCM Biotech, Suzhou, China). The BCA protein detection kit (Beijing Biotech Co., Ltd., China) was used to determine the protein concentration. We added native gel sample loading buffer (NCM Biotech, Suzhou, China) to the protein samples and heated them to denature them. The proteins were separated in gel prepared by ExpressCast PAGE Preparation Kit and then transferred onto 0.45 μm polyvinylidene fluoride (PVDF) membranes (Millipore, Darmstadt, Germany). Then, the membrane was blocked at room temperature for 2 h and incubated with the primary antibody at 4 °C overnight. Then, we used the second antibody (Abmart, Shanghai, China) to incubate the membrane at room temperature for 1 h. Enhanced Chemiluminescent (NCM Biotech, Suzhou, China) was used to show the strip. The primary antibodies we used included: FAM50A Rabbit mAb (ABclonal Technology, Wuhan, China), E-cadherin Polyclonal antibody (Proteintech, Chicago, IL, USA), N-cadherin Rabbit mAb (CST, Boston, MA, USA), Vimentin Polyclonal Antibody (Proteintech, Chicago, IL, USA), β-Tubulin mouse mAb (Abmart, Shanghai, China) and GAPDH antibody (NCM Biotech, Suzhou, China).

### 4.8. Analysis of Cell Proliferation

CCK-8 (DOJINDO, Kumamoto, Kyushu island, Japan) was used to test cell proliferation. The CCK-8 reagent contains WST-8, which can be oxidized to yellow Formazan by dehydrogenase in the cell’s mitochondria. The amount of Formazan is proportional to the number of living cells. Approximately 10,000 cells were cultured in 96-well plates. After the cells were attached, 10 μL of CCK8 reagent was added at 0 h, 12 h and 24 h, then the cells were incubated for 1.5 h. The absorbance at 450 nm was used to react cell activity.

A colony formation assay was also applied to detect the proliferative capacity of cells. We added 800 cells to the six-well plate, then we fixed the cells with 4% paraformaldehyde (Solarbio, Beijing, China) after cell colonies were formed. Crystal violet was used to stain cell colonies.

### 4.9. Drug Sensitivity Test

The IC_50_ assay was used to assess the sensitivity of cells to lenvatinib (APExBIO, Houston, TX, USA). Cells were treated with different concentrations of lenvatinib (0 mg/mL, 2 mg/mL, 4 mg/mL, 8 mg/mL, 16 mg/mL, 32 mg/mL, 64 mg/mL, and 128 mg/mL) for 48 h. The CCK8 kit was used to detect cellular activity. The drc package of the R software was used to analyze data and calculate IC_50_ values. Dose–response curves were visualized using the ggplot2 package.

### 4.10. Analysis of Cell Migration and Invasion

Investigating cell invasion requires the prior addition of matrix gel (Corning, NY, USA) to the transwell chambers (Corning, NY, USA). Cells cultured with serum-free cell culture medium were added to the chambers and medium containing FBS was added to the 24-well plates. About 48 h later, the chambers were fixed with 4% paraformaldehyde and stained with crystal violet.

The transwell chambers were used to research cell migration capacity in the absence of matrigel. Another way to study cell migration is the wound healing assay. Wounds were delimited at the bottom of the plate when cells were fused to 100% in six-well plates. We cultured the cells with serum-free cell medium and took pictures of the wounds using a microscope at 0 h, 12 h, and 24 h.

### 4.11. Flow Cytometric Analysis

Annexin V, FITC Apoptosis Detection Kit (DOJINDO, Kumamoto, Kyushu island, Japan) and Cell Cycle Assay Kit (DOJINDO, Kumamoto, Kyushu island, Japan) were used to examine the cell cycles and apoptosis levels of HCC cells. We treated the cells according to the instructions provided by the supplier and tested them using a flow cytometer (BD Biosciences, Franklin Lakes, NJ, USA).

### 4.12. Immunohistochemistry

IHC was used to detect the protein expression levels in tissues. Subcutaneous xenograft and human tissues were fixed with 4% paraformaldehyde for 24 h. Tissues were dehydrated, embedded in paraffin and sectioned. We processed the tissue sections for dewaxing, hydration, and antigen repair. Then we blocked the tissue sections with serum. Tissue sections were incubated with primary antibody overnight at 4 °C, followed by incubation with secondary antibody for 2 h at room temperature. We observed and photographed the tissues with a microscope. The protein expression was analyzed by ImageJ software.

### 4.13. TUNEL Staining

Dewaxed and hydrated tissue sections were subjected to antigen repair with proteinase K (Servicebio, Beijing, China) working solution at 37 °C. At room temperature, 0.1% triton was used for rupturing cell membranes (Servicebio, Beijing, China). We configured the reaction solution according to the instructions of the TUNEL kit (Servicebio, Beijing, China). Sections were incubated with reaction solution at 37 °C and the cell nucleus was stained with DAPI. The fluorescence intensity was analyzed by ImageJ software.

### 4.14. Mouse Model of Subcutaneous Xenograft Tumor

We fed BALB/c nude mice in a specific-pathogen-free (SPF) environment. FAM50A-shRNA-treated and FAM50A-OE HCCLM3 cells were subcutaneously injected when the mice were at 4 weeks of age. After the tumor reached a certain extent, we anesthetized the mice with isoflurane and took out the tumors. We made all effort to minimize the distress in mice. All animal experiments and procedures were performed according to the relevant laws and regulations of Shanghai Public Health Clinical Center. Our analysis was approved by the ethics committee of Shanghai Public Health Clinical Center.

### 4.15. Statistical Analysis

All experimental results were performed in triplicate. Statistical analyses were performed using the R and Graphpad Prism software. Data analysis was performed using the Student’s *t*-test or one-way ANOVA. *p* < 0.05 was considered statistically significant.

## 5. Conclusions

This study has verified the overexpression of FAM50A in HCC. Patients that highly expressed FAM50A were more likely to have worse prognosis, be more enriched with immune cells, and show a higher stemness degree and a lower ICB response. We demonstrated that the high expression of FAM50A could promote the EMT of HCC cells, reduce sensitivity to lenvatinib and inhibit apoptosis. FAM50A can also promote the proliferation, migration and invasion of HCC cells in vitro. We also validated that the overexpressed FAM50A can increase the growth rate of xenotransplanted tumors in nude mice, inhibit apoptosis and promote proliferation in vivo. In conclusion, we have proven that FAM50A can act as a prooncogenic gene and can serve as a potential diagnostic marker and therapeutic target for HCC. These provide more opportunities for treating HCC.

## Figures and Tables

**Table 1:** Univariate and multivariate COX regression analysis of OS in HCC patients induced by FAM50A.

Characteristics	HR (95% CI) Univariate Analysis	*p* Value	HR (95% CI) Multivariate Analysis	*p* Value
Gender (Male vs. Female)	0.793 (0.557–1.130)	0.2		
Age (>60 vs. <=60)	1.205 (0.850–1.708)	0.295		
Pathologic stage (Stage vs. I~II)	2.504 (1.727–3.631)	<0.001	1.469 (0.200–10.784)	0.705
Histologic grade (G3~4 vs. 1~2)	1.091 (0.761–1.564)	0.636		
T stage (T3~4 vs. 1~2)	2.598 (1.826–3.697)	<0.001	1.643 (0.223–12.104)	0.626
N stage (N1 vs. N0)	2.029 (0.497–8.281)	0.324		
M stage (M1 vs. M0)	4.077 (1.281–12.973)	0.017	1.168 (0.277–4.926)	0.832
Adjacent hepatic tissue inflammation (Mild~Severe vs. None)	1.194 (0.734–1.942)	0.475		
Residual tumor (R1~2 vs. R0)	1.604 (0.812–3.169)	0.174		
Child–Pugh grade (B~C vs. A)	1.643 (0.811–3.330)	0.168		
Vascular invasion (Yes vs. No)	1.344 (0.887–2.035)	0.163		
Tumor status (With tumor vs. Tumor-free)	2.317 (1.590–3.376)	<0.001	2.153 (1.348–3.437)	0.001
AFP (ng/mL) (>400 vs. ≤400)	1.075 (0.658–1.759)	0.772		
Albumin (g/dl) (≥3.5 vs. <3.5)	0.897 (0.549–1.464)	0.662		
Prothrombin time (>4 vs. ≤4)	1.335 (0.881–2.023)	0.174		
Fibrosis ishak score (3/4~5/6 vs. 0~1/2)	0.740 (0.445–1.232)	0.247		
FAM50A (High vs. Low)	2.153 (1.508–3.072)	<0.001	2.272 (1.424–3.627)	<0.001

## Data Availability

The data presented in this study are available on request from the corresponding author.

## Supplementary Materials

The following supporting information can be downloaded at: https://www.mdpi.com/article/10.3390/ijms24043217/s1.

## Abbreviations

HCC	Hepatocellular carcinoma
TIME	Tumor immune microenvironment
AFP	Alpha-fetoprotein
TME	Tumor microenvironment
PBMC	Peripheral Blood Mononuclear Cells
CLL	Chronic Lymphoblastic Leukemia
TCGA	The Cancer Genome Atlas
GEO	Gene Expression Overview
UALCAN	University of Alabama at Birmingham Cancer data analysis Portal
TIMER	Tumor Immune Estimation Resource
ROC	Receiver Operating Characteristic
AUROC	Area Under ROC
CI	Confidence Interval
OS	Overall Survival
DSS	Disease-Specific Survival
OCLR	One-class logistic regression
ICB	Immune Checkpoint Blockade
TIDE	Tumor Immune Dysfunction and Exclusion
shRNA	short hairpin RNA
qRT-PCR	Quantitative Real-Time Polymerase Chain Reaction
EMT	Epithelial–Mesenchymal Transition
VEGFR	Vascular Endothelial Growth Factor Receptors
IC_50_	Half-maximal inhibitory concentration
FCM	Flow cytometry
ECM	Extracellular matrix
BMM	Basement Membrane Matrix
IHC	Immunohistochemistry
IF	Immunofluorescence
FITC	Fluorescein isothiocyanate
NAFLD	Nonalcoholic fatty liver disease
CPTAC	Clinical Proteomic Tumor Analysis Consortium
DMEM	Dulbecco’s modified Eagle’s medium
FBS	Fetal Bovine Serum
FPKM	Fragments Per Kilobase Million
TPM	Transcripts Per Million
PVDF	Polyvinylidene fluoride
SPF	specific-pathogen-free
