# Supplementary material for: Proto-Oncogene FAM50A Can Regulate the Immune Microenvironment and Development of Hepatocellular Carcinoma In Vitro and In Vivo
Source: Int J Mol Sci. 2023 Feb 6;24(4):3217. doi: 10.3390/ijms24043217 (PMC9966472; doi:10.3390/ijms24043217)
Supplement: Supplementary file 1 [file ijms-24-03217-s001.zip › ijms-2143459-supplementary file.pdf]

Supplementary Materials

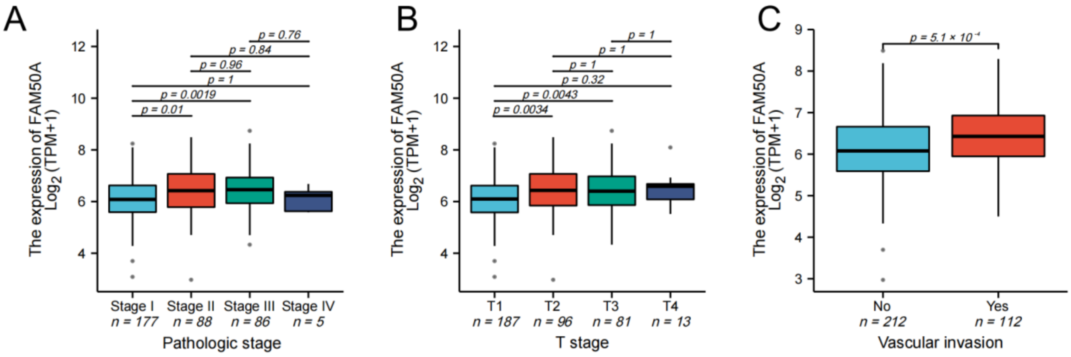

Figure S1. Different expression of FAM50A in various subtypes of HCC patients. (**A**) FAM50A expression in patients with different pathologic stages. (**B**) FAM50A expression in patients with different T stages. (**C**) FAM50A expression in patients with vascular invasion or not.

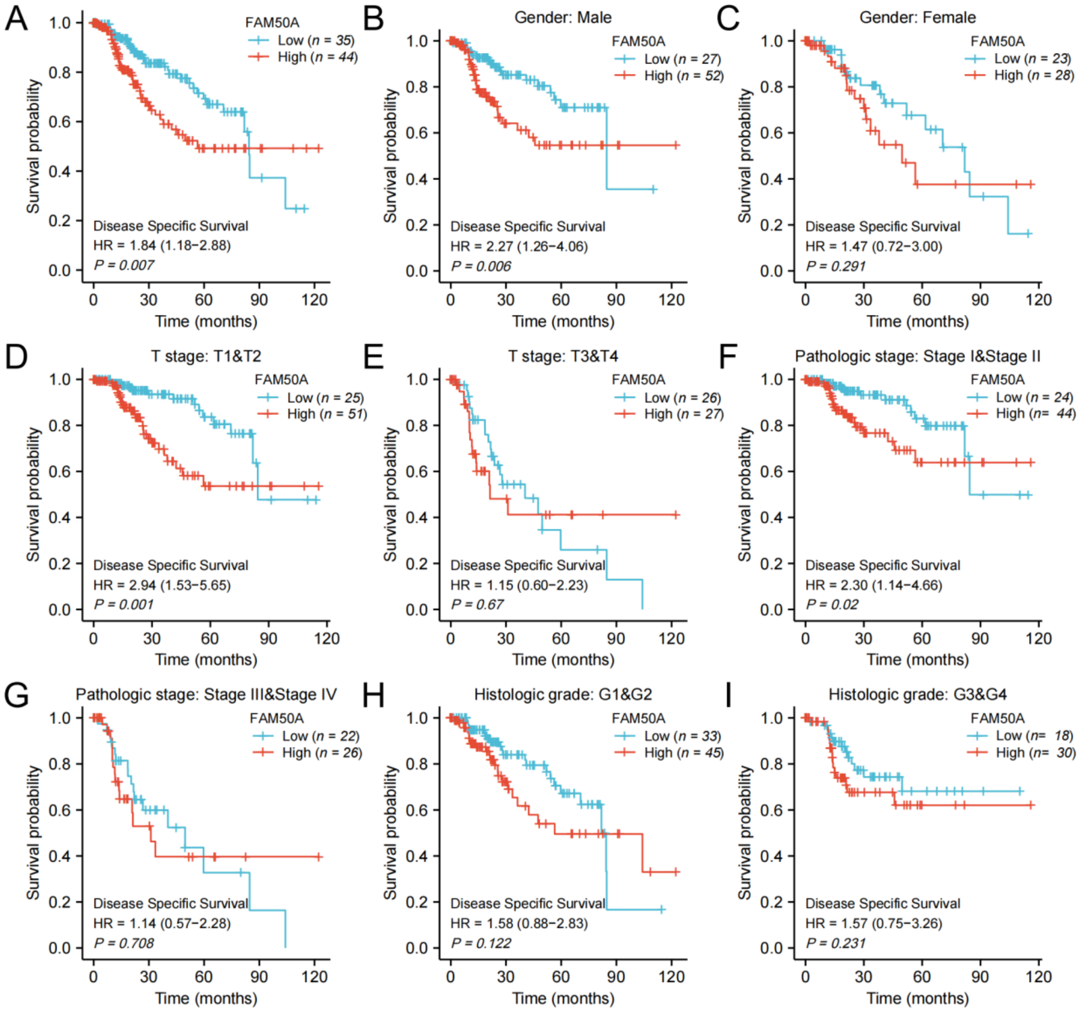

Figure S2. K-M survival analysis. We analyzed the effects of FAM50A on DSS in HCC patients with the help of information from TCGA. (**A**) All HCC patients. (**B**) Male patients. (**C**) Female patients. (**D**) T stage (T1 & T2). (**E**) T stage (T3 & T4). (**F**) Pathologic stage I&II. (**G**) Pathologic stage III&IV. (**H**) Histologic grade 1&2. (**I**) Histologic grade 3&4.

Table S1. Univariate and multivariate cox regression analysis of DSS in HCC patients induced by FAM50A.

**Characteristics**	**HR (95% CI) Univariate analysis**	**P value**	**HR (95% CI) Multivariate analysis**	***P value***
Gender (Female vs. Male)	0.813 (0.516–1.281)	0.373		
Age (<=60 vs. >60)	0.846 (0.543–1.317)	0.458		
Pathologic stage (Stage I~II vs. III~IV)	3.803 (2.342–6.176)	<0.001	0.925 (0.093–9.199)	*0.947*
Histologic grade (G1~2 vs. 3~4)	1.086 (0.683–1.728)	0.726		
T stage (T1~2 vs. 3~4)	3.639 (2.328–5.688)	<0.001	2.577 (0.250–26.556)	*0.426*
N stage (N0 vs. N1)	3.612 (0.870–14.991)	0.077		
M stage (M0 vs. N1)	5.166 (1.246–21.430)	0.024	2.567 (0.454–14.511)	*0.286*
Adjacent hepatic tissue inflammation (None vs. Mild~Severe)	1.403 (0.768–2.566)	0.271		
Residual tumor (R0 vs. 1~2)	1.678 (0.728–3.870)	0.224		
Child-Pugh grade (A vs. B~C)	2.560 (1.123–5.834)	0.025	3.671 (1.100–12.243)	*0.034*
Vascular invasion (No vs. Yes)	1.277 (0.707–2.306)	0.418		
AFP (ng/mL) (<=400 vs. >400)	0.867 (0.450–1.668)	0.668		
Albumin (g/dL) (<3.5 vs. >=3.5)	1.148 (0.586–2.250)	0.687		
Prothrombin time (<=4 vs. >4)	1.778 (1.054–2.999)	0.031	1.734 (0.650–4.626)	*0.272*
Fibrosis ishak score (0~1/2 vs. 3/4~5/6)	0.660 (0.340–1.279)	0.218		
FAM50A (Low vs. High)	1.841 (1.178–2.878)	0.007	2.948 (1.257–6.910)	*0.013*

Table S2. Correlation between FAM50A and immune cell markers in HCC.

**Description**	**Gene markers**	**R**	***p***
Macrophage	CD68	0.3	1.3×10^{-12}
	CD11b (ITGAM)	0.3	8.8×10^{-13}
CD4+ T cell	AIM2	0.22	3×10^{-7}
	CCL4	0.13	2.4×10^{-3}
	CCNB1	0.48	*0*
	EXO1	0.53	*0*
	KIF11	0.45	*0*
	KNTC1	0.36	*0*
	NUF2	0.47	*0*
	PRC1	0.48	*0*
	RTKN2	0.31	1.4×10^{-13}
CD8+T cell	CD8A	0.18	3.8×10^{-5}
	CD8B	0.22	2.9×10^{-7}
Dendritic cell	HLA-DPB1	0.29	1.4×10^{-11}
	HLA-DQB1	0.22	3.1×10^{-7}
	HLA-DRA	0.27	5.7×10^{-10}
	HLA-DPA1	0.25	1×10^{-8}
B cell	CD20(KRT20)	0.13	2×10^{-3}
Neutrophils	CD11b (ITGAM)	0.3	8.8×10^{-13}
	CD15(FUT4)	0.19	1.4×10^{-05}
